# Supplementary material for: Recurrence and propagation of past functions through mineral facilitated horizontal gene transfer
Source: Front Microbiol. 2024 Nov 7;15:1449094. doi: 10.3389/fmicb.2024.1449094 (PMC11580795; doi:10.3389/fmicb.2024.1449094)
Supplement: Supplementary file 1 [file Data_Sheet_1.pdf]

SUPPORTING INFORMATION

Supporting section 1: minerals

1.1: Adsorption and mineral data

FTIR spectra of synthesized goethite shows peaks characteristic of Fe-O-OH bonds, confirming its purity.

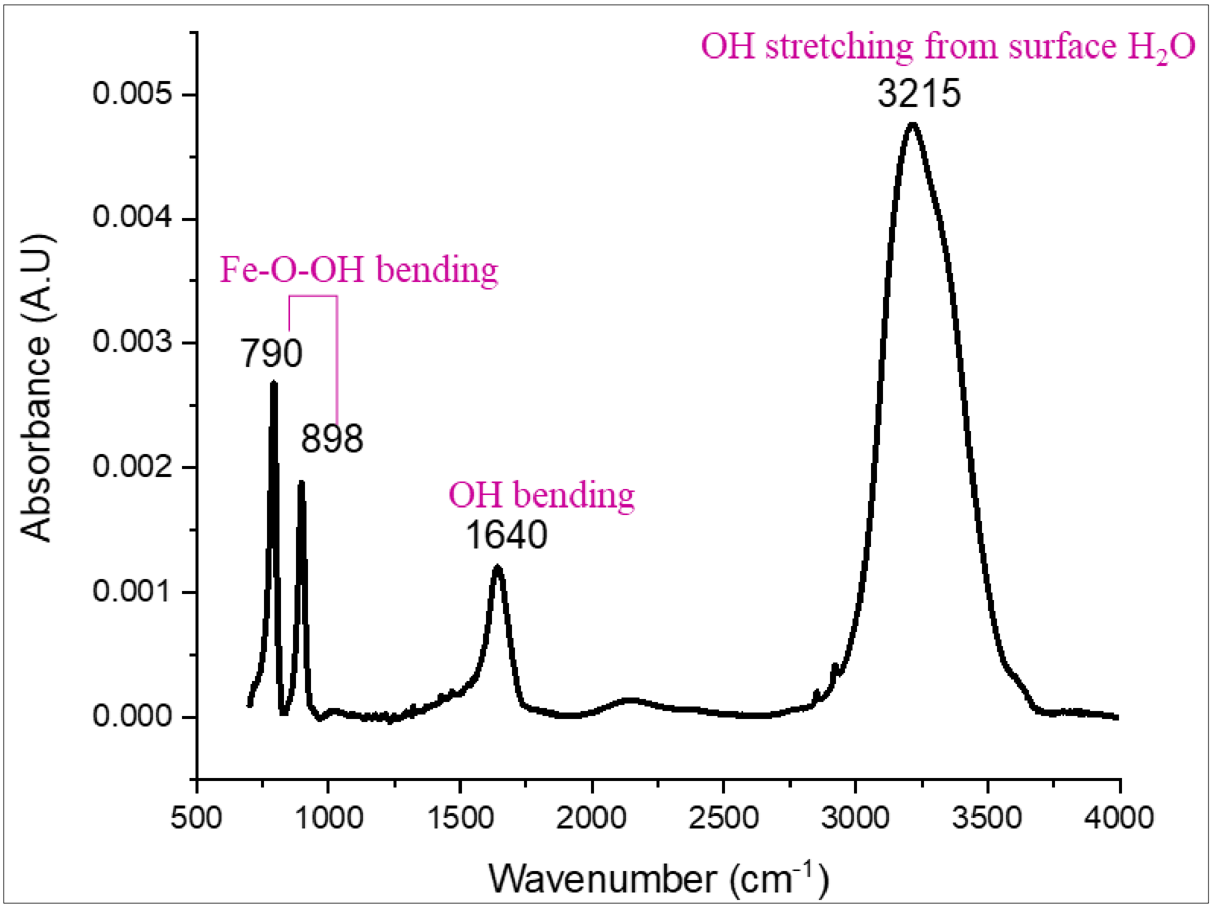

Figure S1.1: FTIR spectra of the goethite nanoparticles. The most prominent peaks in the spectra along with their contributions are indicated. Assignments are taken from Chukanov, N.V 2014 (Chukanov, 2014).

Table S1.1: Properties of the minerals used in the study.

Mineral	Type	Specific surface area (SSA) $m^2\ g^{-1}$	Surface area of the minerals used for HGT (m^2) *	Active sites per nm^2 (as obtained from references)	Active sites on the total surface area used for HGT (nm^2) **
**Hematite**	Iron oxy-hydroxide	108.5	0.1	6.36 (García et al., 2021)	$0.636*10^{18}$
**Goethite**		90.1	0.06	5.5 (Villalobos et al., 2003)	$0.33*10^{18}$
**Kaolinite**	Clay minerals	11.7	0.1	0.25 (Sanders et al., 2010)	$0.025*10^{18}$
**Mica**		1.5	0.06	5 (Brugman et al., 2020)	$0.3*10^{18}$
**Calcite**	Carbonate	0.31	0.06	8.5 (Pokrovsky et al., 2000)	$0.51*10^{18}$
**Quartz**	Non-clay silicate	1.5	0.06	4 (Tang et al., 2015)	$0.24*10^{18}$

* *Calculation details for HGT surface area*

Hematite: SSA = 108.5 m^2, Amount weighed for HGT = 1.5 mg, SA_{HGT} = (108.5*1.5)/1000 mg = **0.1 m^2**
Goethite: SSA= 90.1 m^2, Amount weighed for HGT= 0.7 mg, SA_{HGT} = (90.1*0.7)/1000 mg = **0.06 m^2**
Kaolinite: SSA= 11.7 m^2, Amount weighed for HGT= 10 mg, SA_{HGT} = (11.7*10)/1000 mg = **0.1 m^2**
Mica: SSA= 1.5 m^2, Amount weighed for HGT= 40 mg, SA_{HGT} = (1.5*40)/1000 mg = **0.06 m^2**
Calcite: SSA = 0.31 m^2, Amount weighed for HGT= 200 mg, SA_{HGT} = (0.31*200)/1000 mg = **0.06 m^2**
Quartz: SSA = 1.5 m^2, Amount weighed for HGT= 40 mg, SA_{HGT} = (40*1.5)/1000 mg = **0.06 m^2**

** *Calculation details for number of active sites*

Hematite: No. of active sites present per nm^2 = 6.36,
Total no. of active sites present in 0.1 m^2 = $6.36*0.1*10^{18}$ = **$0.636 * 10^{18}$**
Goethite: No. of active sites present per nm^2 = 5.5,
Total no. of active sites present in 0.06 m^2 = $5.5*0.06*10^{18}$ = **$0.33 * 10^{18}$**
Kaolinite: No. of active sites present per nm^2 = 0.25,
Total no. of active sites present in 0.1 m^2 = $0.25*0.1*10^{18}$ = **$0.025 * 10^{18}$**
Mica: No. of active sites present per nm^2 = 5,
Total no. of active sites present in 0.06 m^2 = $5*0.06*10^{18}$ = **$0.3* 10^{18}$**
Calcite: No. of active sites present per nm^2 = 8.5,
Total no. of active sites present in 0.06 m^2 = $8.5*0.06*10^{18}$ = **$0.51 * 10^{18}$**
Quartz: No. of active sites present per nm^2 = 4,
Total no. of active sites present in 0.06 m^2 = $4*0.06*10^{18}$ = **$0.24 * 10^{18}$**

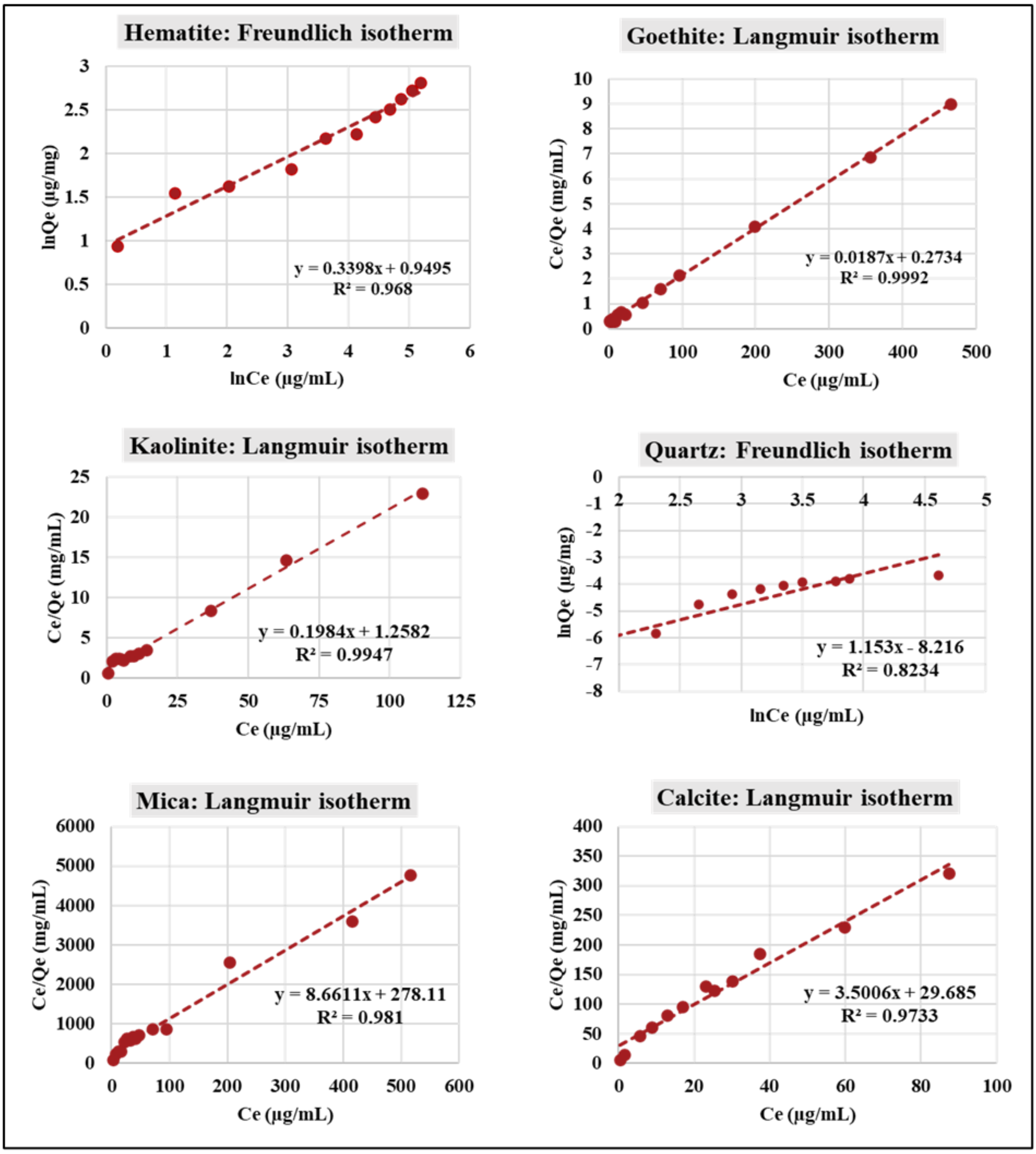

Figure S1.2: Adsorption isotherm plots for DNA binding on minerals. Adsorption data for salmon sperm DNA and the different minerals were used to fit either the linear Langmuir equation for goethite, kaolinite, mica and calcite or the linear Freundlich equation for hematite and quartz. Red dots represent the adsorption data points and the dotted line refers to Langmuir or Freundlich fit. 'Ce' refers to the equilibrium DNA concentration and 'Qe' is the adsorbed DNA concentration. The data are representative of three experiments and are plotted as mean.

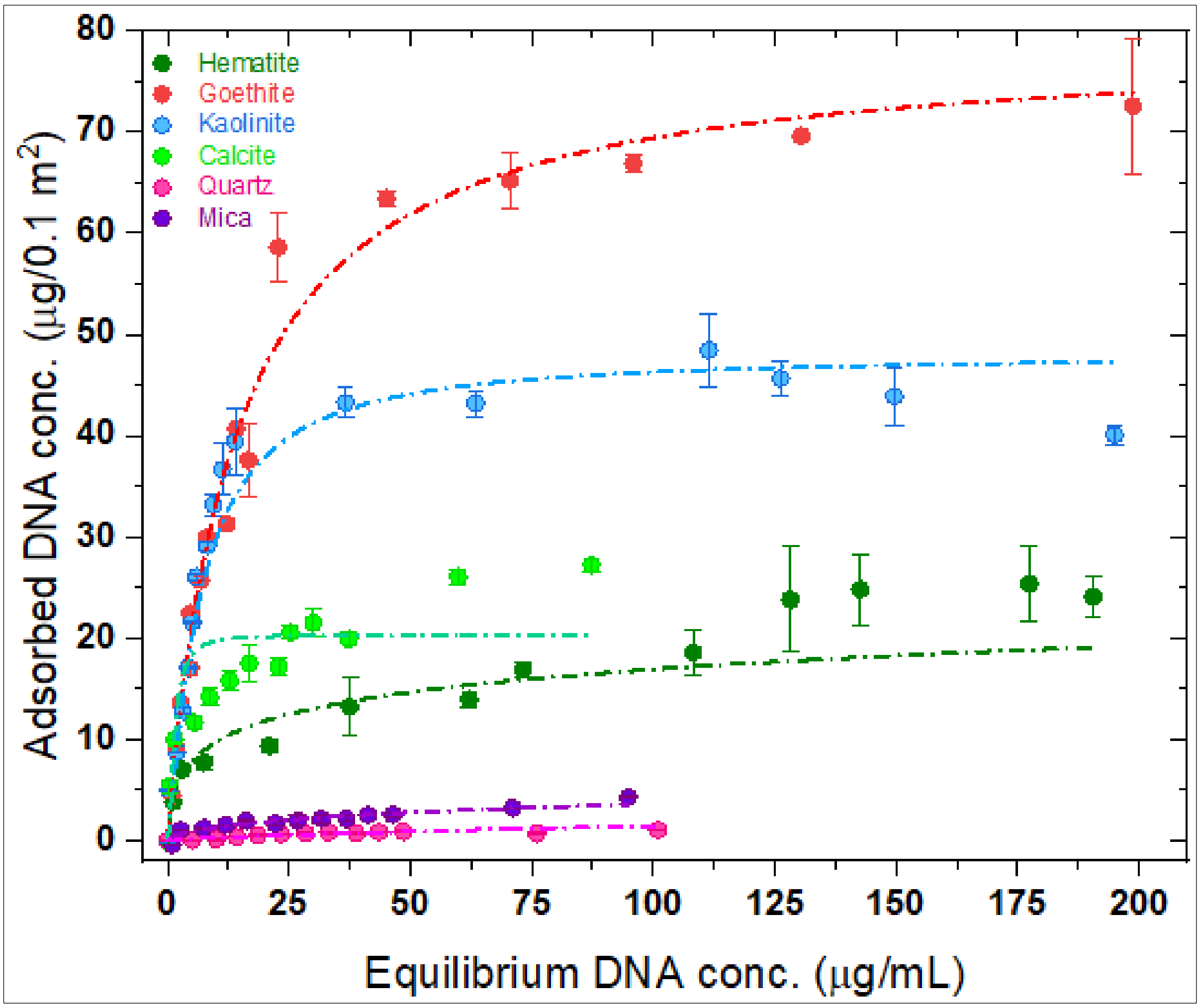

Figure S1.3: Equilibrium adsorption of salmon sperm DNA corresponding to 0.1 m^2 of the mineral surface area in saline under physiological conditions. Serially increasing concentrations of salmon sperm DNA (0-200 µg/mL) were mixed with sonicated mineral suspensions in 150 mM NaCl at pH 7. After incubation for 16 hrs, the amount of adsorbed DNA was estimated using absorbance at 260 nm for the indicated minerals. Langmuir or Freundlich fitting was performed to generate the adsorption isotherms for each DNA-mineral pair. (Langmuir Fitting Goethite, kaolinite, calcite, mica; Freundlich fitting: Hematite and quartz). The data are representative of three experiments and are plotted as mean ±S.D.

1.2 Information on mineral morphologies and topographies

A crystal face represents a flat, external surface of a crystal that forms naturally, as the crystal grows. It reflects the internal symmetry and structure of the crystal, corresponding to specific planes of atoms in the crystal lattice. Further, orientation of weak chemical bonds along a specific crystal plane produces a plane of weakness (cleavage plane) inside a mineral along which the mineral preferentially breaks macroscopically, known as cleavage. Cleavage planes as well as crystal faces are oriented along specific crystallographic directions and named using Miller indices.

For example, the (001) crystal phase represents the basal plane in mica. Mica and kaolinite are both clay minerals and have permanent negatively charged basal planes and positively charged edge sites (Christenson and Thomson, 2016), (Kumar et al., 2017). In contrast to mica, kaolinite has two types of basal planes where one is composed of Al-octahedra, which are slightly positive in our experiments. The basal planes are atomically flat. For calcite, the (104) crystal face is the most abundant in nature and the one represented in our experiments. The local topography on the calcite surface is composed of atomically flat terraces and the surface contains obtuse and acute step edges which carry a stronger positively charge density than the terraces (Wolthers et al., 2012). Hematite and goethite are nano rods composed of several crystal faces such as (100), (110) and (021) with slightly distinct charges. In general, goethite displays a higher density of hydroxyl groups and positive charges than hematite (Barrón and Torrent, 1996).

Table S1.2: Mineral weights and DNA concentrations used in the study.

**Mineral type**	**Weight of the mineral (mg)**	**DNA added (µg µL^{-1})**	**DNA adsorbed (µg mineral wt^{-1})**	**Adsorption parameters**		
				**Langmuir adsorption**		
				**Q_{max}**	**K_L**	**r^2**
Goethite	0.7	0.02	11±2.6	53.5	0.07	0.99
Kaolinite	10	0.015	11±2	5.04	0.16	0.99
Mica	40	0.075	1.3±0.6	0.11	0.03	0.98
Calcite	200	0.015	9.3±1.6	0.3	0.12	0.97
				**Freundlich adsorption**		
				**K_F**	**n**	**r^2**
Hematite	1.5	0.03	8.3±1.1	2.58	2.94	0.97
Quartz	40	0.05	1.3±0.6	3700	0.86	0.82

Q_{max}: maximum adsorption capacity (µg/mg), K_L: Langmuir constant, K_F: Freundlich constant, n: Freundlich exponent

Supporting section 2: Cell viability and bacterial transformation

To study the effects of minerals on bacterial viability, we exposed *A. baylyi* to fixed mineral concentrations (see Table S1.2 for concentrations) in saline, M9 supplemented with tryptophan or LB

for a period of 2 hrs under shaking conditions. The mineral-bacterial suspensions were then plated on M9 supplemented with tryptophan, incubated at 30^0C for 48 hrs and the colonies were counted. Except for goethite and hematite, no significant reduction in the cell viability was observed for the minerals.

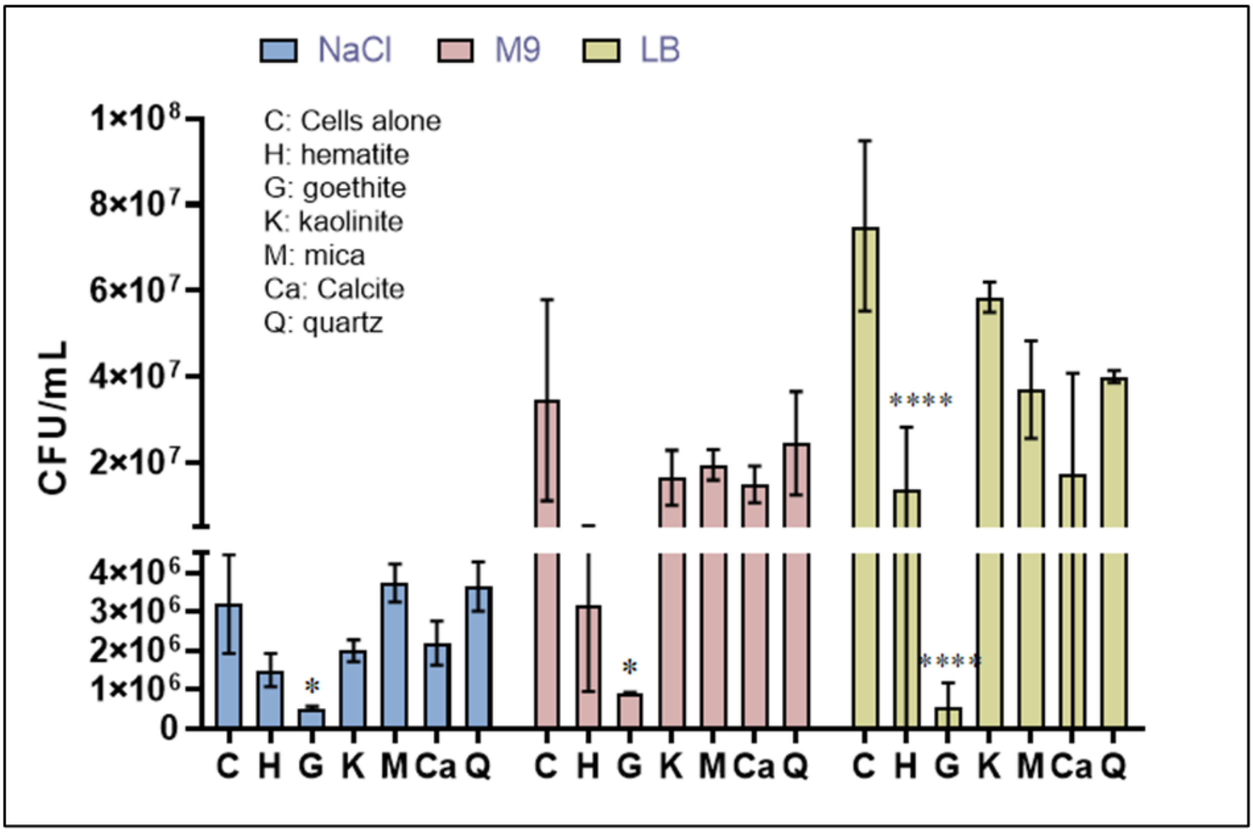

Figure S2.1: Effect of minerals on cell viability of *A.baylyi*. Competent *A. baylyi* cells were exposed to the various minerals as indicated for a period of 2 hrs in either saline (150 mM NaCl), M9 supplemented with tryptophan or LB medium. Post exposure, the cells were plated onto M9 supplemented with 50 mg/L tryptophan and incubated at 30^OC for 48 hrs. Colonies were counted and reported as CFU/mL. The data are representative of three biological replicates and are plotted as mean ± S.D. For statistical analysis, two-way ANOVA was performed for all minerals with respect to cells alone (For eg.: Cells alone vs cells in hematite grown in NaCl.)

2.1 Relationship between transformation efficiency and number of active sites

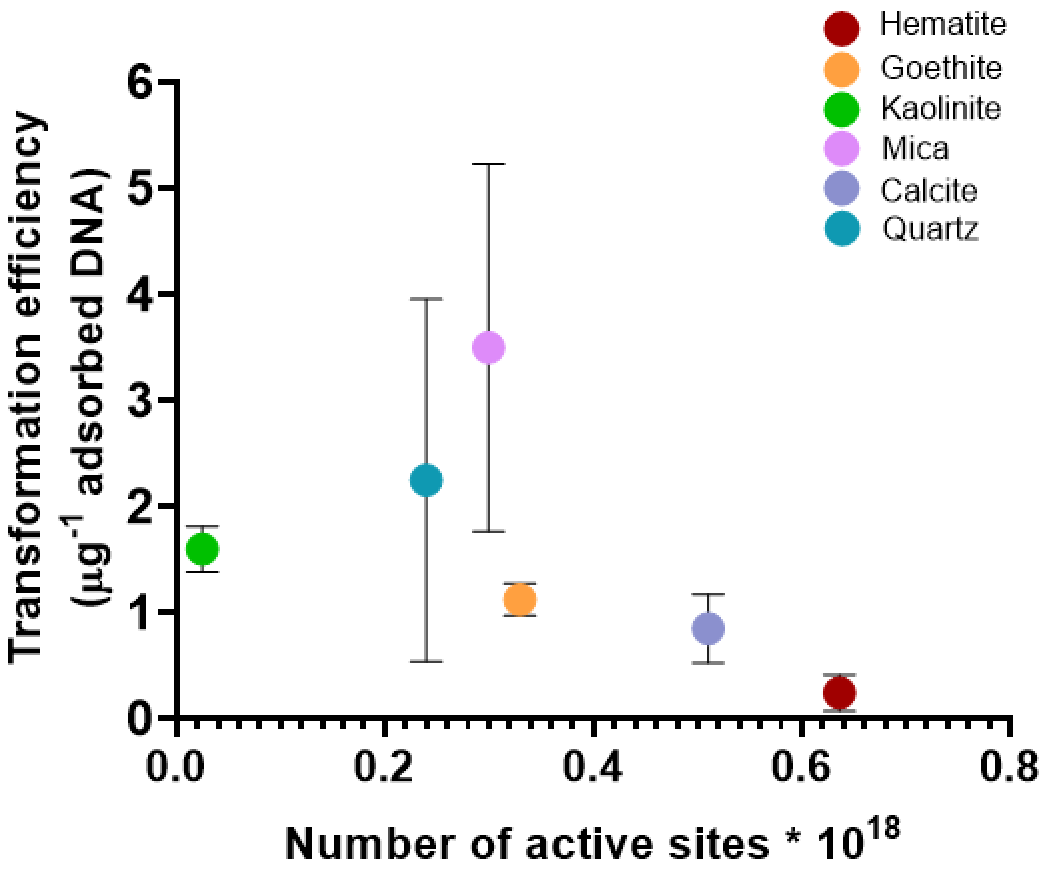

Figure S2.2: Transformation efficiency as a function of active site density of the different minerals.

Supporting section 3. AFM

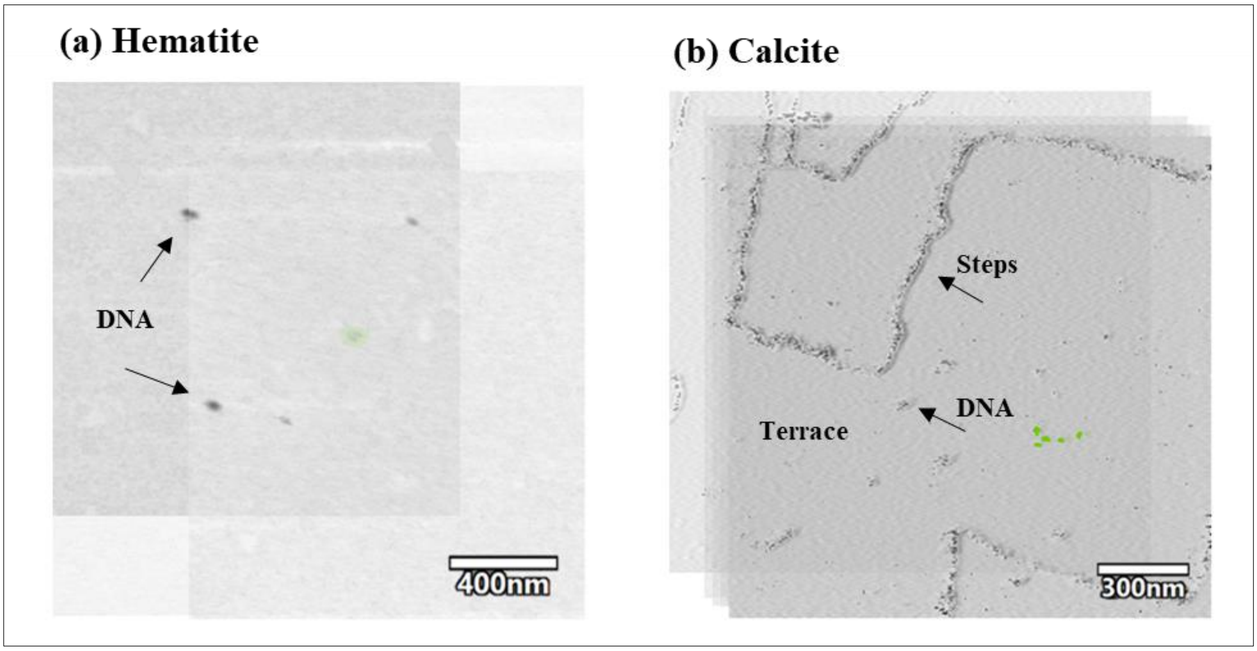

Figure S3.1: Time-dependent tracking of DNA using AFM image stacks of hematite and calcite. (a) Overlay of 6 hematite images taken during a time span of 6 minutes while tracking 5 DNA molecules (DNA molecules are highlighted as black dots); (b) Overlay of 6 calcite images taken during a time span of 6 minutes while tracking 5 individual DNA molecules anchored to a terrace.

Images in the TIFF format were retrieved **from AFM movies using software Igor Pro v 6.38 and** processed using MATLAB. In MATLAB, hematite images pixel values were converted into double data type and then the negative images were obtained by subtracting the scaled pixel values to 1. After this step, the background becomes light gray and the particles on the surface appear as black dots. A mask was applied to remove most of the particles (black dots) in the images. The mask assigned the average background pixel value to all the pixels with a value higher than 0.89 except in 5 specific locations in the images (those corresponding to the particles of interest). Calcite images were also processed in MATLAB and their pixel values were converted into double data type. In this case, instead of obtaining the negative image, the background features were highlighted by using a mask. This mask assigned a value of 0 to all the pixels that had values higher than a number in the range from 0.75 to 0.9. The selection of a value within this range depended on the overall pixel values in the image: images with lower pixel values required a lower value for the mask and images with higher pixel values required a higher value. After this, the processed images were exported in TIFF format and superimposed using the background features as guides in PowerPoint. The DNA molecules in the images (highlighted as black dots) were tracked throughout the images and their area of movement was coloured green.

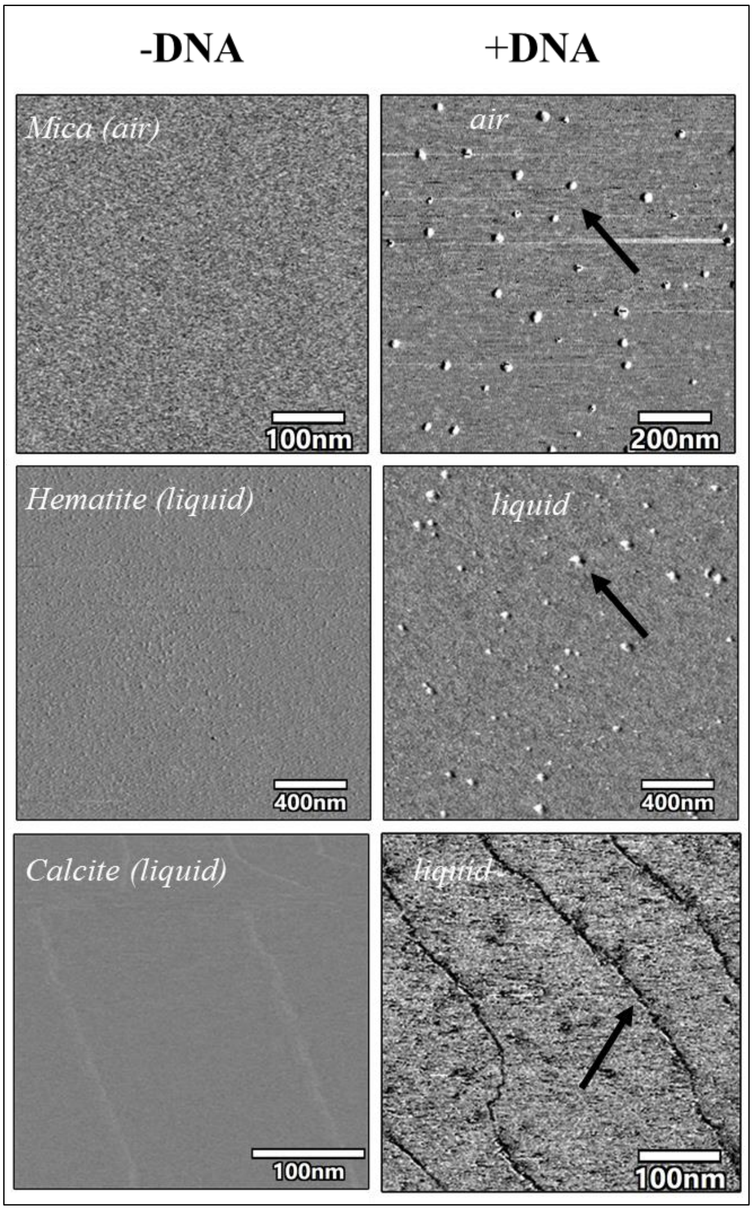

Figure S3.2: AFM phase images confirm the presence and localisation of DNA molecules on the mineral surface. DNA on the minerals appears as spots with different greyscale in contrast to the mineral surface. The black arrows indicate the localisation of DNA.

References

- Barrón, V., and Torrent, J. (1996). Surface Hydroxyl Configuration of Various Crystal Faces of Hematite and Goethite. Journal of Colloid and Interface Science 177(2), 407-410. doi: https://doi.org/10.1006/jcis.1996.0051.
- Brugman, S.J.T., Werkhoven, B.L., Townsend, E.R., Accordini, P., van Roij, R., and Vlieg, E. (2020). Monovalent – divalent cation competition at the muscovite mica surface: Experiment and theory. *Journal of Colloid and Interface Science* 559**,** 291-303. doi: https://doi.org/10.1016/j.jcis.2019.10.009.
- Christenson, H.K., and Thomson, N.H. (2016). The nature of the air-cleaved mica surface. Surface Science Reports 71(2), 367-390. doi: https://doi.org/10.1016/j.surfrep.2016.03.001
- Chukanov, N.V. (2014). "IR Spectra of Minerals and Reference Samples Data," in *Infrared spectra of mineral species: Extended library*. (Dordrecht: Springer Netherlands), 21-1701
- García, D., Lützenkirchen, J., Huguenel, M., Calmels, L., Petrov, V., Finck, N., et al. (2021). Adsorption of Strontium onto Synthetic Iron(III) Oxide up to High Ionic Strength Systems. *Minerals* 11(10)**,** 1093.
- Kumar, N., Andersson, M.P., van den Ende, D., Mugele, F., and Siretanu, I. (2017). Probing the Surface Charge on the Basal Planes of Kaolinite Particles with High-Resolution Atomic Force Microscopy. Langmuir 33(50), 14226-14237. doi: 10.1021/acs.langmuir.7b03153
- Pokrovsky, O.S., Mielczarski, J.A., Barres, O., and Schott, J. (2000). Surface Speciation Models of Calcite and Dolomite/Aqueous Solution Interfaces and Their Spectroscopic Evaluation. *Langmuir* 16(6)**,** 2677-2688. doi: 10.1021/la980905e.
- Sanders, R.L., Washton, N.M., and Mueller, K.T. (2010). Measurement of the Reactive Surface Area of Clay Minerals Using Solid-State NMR Studies of a Probe Molecule. *The Journal of Physical Chemistry C* 114(12)**,** 5491-5498. doi: 10.1021/jp906132k.
- Tang, C., Zhu, J., Li, Z., Zhu, R., Zhou, Q., Wei, J., et al. (2015). Surface chemistry and reactivity of SiO2 polymorphs: A comparative study on α-quartz and α-cristobalite. *Applied Surface Science* 355**,** 1161-1167. doi: https://doi.org/10.1016/j.apsusc.2015.07.214.
- Villalobos, M., Trotz, M.A., and Leckie, J.O. (2003). Variability in goethite surface site density: evidence from proton and carbonate sorption. *Journal of Colloid and Interface Science* 268(2)**,** 273-287. doi: https://doi.org/10.1016/j.jcis.2003.07.044.
